# Effects of various supplemental levels of multi-enzyme complex on amino acid profiles in egg yolk, antioxidant capacity, cecal microbial community and metabolites of laying hens

**DOI:** 10.3389/fmicb.2024.1466024

**Published:** 2024-11-28

**Authors:** Qixin Huang, Rui Chen, Wenzi Wu, Jinghui Fan, Xin Ma, Zhou Chen, Wenxin Ye, Lichun Qian

**Affiliations:** ^1^Key Laboratory of Animal Nutrition and Feed Science in East China, Ministry of Agriculture, College of Animal Sciences, Zhejiang University, Hangzhou, China; ^2^Hainan Institute of Zhejiang University, Sanya, China; ^3^Hangzhou Academy of Agricultural Sciences, Hangzhou, China

**Keywords:** enzymes, microbes, metabolites, laying hens, amino acid profiles in yolk

## Abstract

This study aimed to investigate the effects of multi-enzyme (alkaline protease, xylanase, glucanase, β-mannanase, cellulase, acid protease, glucoamylase, and α-galactosidase) on antioxidant capacity, egg quality, amino acid profiles in yolk, cecal microflora and metabolites in laying hens. A total of 384 Jingfen No.6 laying hens aged 65 weeks were randomly divided into 4 treatments groups (6 replicates per group) and fed diets containing 0, 150, 300, or 600 mg kg^−1^ multi-enzyme over an 8-week feeding duration. Our findings revealed that supplementation with 600 mg kg^−1^ of multi-enzyme significantly increased the albumen height (*P* < 0.05) and haugh unit (*P* < 0.05). Moreover, as the levels of multi-enzyme supplementation in the diet increased, there were significant increases in activities of total antioxidant capacity (T-AOC) in serum (*P* < 0.05) and glutathione peroxidase (GSH-Px) in the liver (*P* < 0.05). Different levels of multi-enzyme supplementation significantly affected the composition of amino acid profiles in the yolk. Furthermore, the results from 16S rRNA sequencing and untargeted metabolomics analysis of cecal content revealed that multi-enzyme supplementation altered the cecal microflora and metabolite profiles. We found the relative abundance of the *Bacteroidota* phyla in T600 group was significantly increased (*P* < 0.05) compared to CON and T150 groups, but the relative abundance of the *Firmicutes* phylum in T600 group were significantly lower than T150 group (*P* < 0.05). At the genus level, the relative abundance of the *Parabacteroides* genera in T300 group, the *Faecalibacterium* genera in T300 and T600 groups, the *norank_f_Prevotellaceae* genera in treatment groups (T150, T300 and T600), the *norank_f_Peptococcaceae* genera in T600 group, and the *Monoglobus* genera in T1 group were significantly increased. The KEGG pathway analysis showed that the common enrichment metabolic pathways of each treatment group compared to the CON group were glycine, serine and threonine metabolism, foxo signaling pathway and mTOR signaling pathway, and the enrichment metabolic pathways shared by T300 vs CON and T600 vs CON was galactose metabolism and glycolysis/gluconeogenesis pathways. Correlation analysis identified notable relationships between specific microbes and metabolites with T-AOC in serum, GSH-Px activity in the liver, amino acids in yolk, albumen height, and haugh units. Overall, this study suggests that multi-enzyme supplementation regulated the cecal microbial community and metabolism, potentially influencing amino acid profiles in yolk, antioxidant capacity, and egg quality.

## 1 Introduction

Given the escalating costs of soybeans as a primary feed ingredient and their constrained availability, the poultry industry faces an urgent need to explore novel, safe, and efficient alternatives to traditional feed additives. This exploration is crucial for reducing feed expenses and improving profit margins. Currently, several alternative protein sources have emerged as viable options, including cottonseed meal (da Silva et al., 2021; Tarnonsky et al., 2023) and rapeseed meal (Czech et al., 2021; Pirgozliev et al., 2022).

Cottonseed meal, a by-product of oil extraction from shelled cottonseed, contains a crude protein content ranging from 34 to 40% (Zhang et al., 2018), which is comparable to that of soybean meal. Similarly, rapeseed meal, a by-product of rapeseed oil extraction (Kasprzak et al., 2016), has a crude protein content exceeding 33%, accompanied by a well-balanced amino acid composition and sufficient levels of available lysine (Lomascolo et al., 2012). Furthermore, both of these vegetable protein sources are economically advantageous and readily available, making them highly favored in the agricultural sector. However, the elevated crude fiber content inherent in both cottonseed and rapeseed meals reduces their feed-utilization efficiency in poultry farming, necessitating supplementation with feed enzymes (da Silva et al., 2021; Wiśniewska et al., 2023).

Currently, the addition of enzymes to poultry feed to enhance digestion and absorption is common practice (Yi et al., 2023). Previous studies have indicated that soybean meal, rapeseed meal and cottonseed meal are plant-based protein ingredients that contain large amounts of non-starch polysaccharides (NSPs), including mannose, xylose, galactose, and arabinose (Lomascolo et al., 2012; Knudsen, 2014; Yi et al., 2024a). However, animals lack specific endogenous enzymes capable of degrading NSPs, necessitating the inclusion of NSP-degrading enzymes, known as carbohydrases, in feed formulations. Jasek et al. (2018) reported that supplementing broiler diets with a multi-carbohydrase formulation containing α-galactosidase and xylanase inclusion can enhance nutrient and ileal amino acid digestibility across various dietary nutrient profiles. Musigwa et al. (2021) demonstrated that the addition of supplemental multi-carbohydrase enhanced dietary energy utilization regardless of dietary crude protein concentration. Several studies have indicated that the protein in diet may not be completely digested by endogenous proteases as they traverse the gastrointestinal tract (Hu et al., 2020). Consequently, exogenous proteases are commonly supplemented in animal diets to enhance protein utilization. Walk et al. (2018) demonstrated that novel protease supplementation could improve the apparent ileal amino acid digestibility. Previous research has shown that dietary supplementation with various levels of protease DE200 significantly enhances the production performance of laying hens while reducing feed conversion ratios (Cai et al., 2024).

Although numerous studies have explored the individual applications of carbohydrases or exogenous proteases in poultry production, few have investigated the synergistic effects of their combined use. Therefore, this study aims to examine the impact of multi-enzyme formulation (alkaline protease, xylanase, glucanase, β-mannanase, cellulase, acid protease, glucamylase and α-galactosidase) on the antioxidant capacity, egg quality, cecal microbiota and metabolites of laying hens.

## 2 Materials and methods

### 2.1 Animal ethics

All the experimental procedures conducted in this study were ethically approved by the Institutional Animal Care and Use Committee of Zhejiang University (ZJU20240150).

### 2.2 Enzyme preparation

The enzyme utilized in this study was a commercial preparation sourced from Wuhan Sunhy Biology Co., Ltd (Hubei, China). This multi-enzyme preparation comprised the following activities per gram: 129,600 U of alkaline protease (produced by *Bacillus subtilis*), 30,000 U of xylanase (produced by *Pichia pastoris*), 3,500 U of glucanase (produced by *Pichia pastoris*), 1,000 U of β-mannanase (produced by *Pichia pastoris*), 1,500 U of cellulase (produced by *Trichoderma reesei*), 1,000 U of acid protease (produced by *Bacillus subtilis*), 3,000 U of glucamylase (produced by *Trichoderma reesei*), and 200 U of α-galactosidase (produced by *Pichia pastoris*).

### 2.3 Animal management and experimental design

A total of 384 Jingfen No.6 laying hens, aged 65 weeks, were randomly assigned to four treatment groups, each consisting of six replicates. Within each replicate, 16 birds were housed in cages containing four hens per cage, across an 8-week feeding period. The four dietary treatments included: a basal diet (CON), the basal diet supplemented with 150 mg kg^–1^ of multi-enzyme (T150), the basal diet supplemented with 300 mg kg^–1^ of multi-enzyme (T300), and the basal diet supplemented with 600 mg kg^–1^ of multi-enzyme (T600). The selection of the 150, 300, and 600 mg kg^–1^ multi-enzyme levels were conducted under the guidance of the technical staff of Wuhan Sunhy Biology Co. Ltd (Hubei, China) company. Details about each single enzyme product are available on their website.^1^ The feeding regimen was determined in accordance with the “Chicken Feeding Standard NY/T33-2004.” The nutritional composition of the basal diet is detailed in Table 1, the cottonseed meal in the feed is dephenolized and all diets were administered in mash form. In this experiment, a stepwise blending method was used to add trace amounts of the multi-enzyme to the feed.

**TABLE 1:** Ingredients and nutrient contents of the basal diet (as-fed basis).

Items	Value
**Ingredients, %**	
Corn	65.50
Soybean meal	11.50
Dephenolized cottonseed meal	6.00
Rapeseed meal	6.00
Limestone powder	7.00
Premix^1^	4.00
Total	100.00
**Nutrient composition**	
Metabolism energy, Mcal/kg	2.62
Crude protein^2^	16.57
Crude fiber^2^	2.50
Crude ash^2^	9.70
Ether extract^2^	3.00
Methionine	0.36
Lysine	0.86
Calcium	2.97
Total phosphorus	0.46

^1^Premix provided the following per kilogram of diet: vitamin A, 10000 IU; vitamin D3, 1800 IU; vitamin E, 10 IU; vitamin K, 10 mg; vitamin B12, 1.25 mg; thiamine, l mg; riboflavin, 4.5 mg; calcium pantothenate, 50 mg; NaCl, 3g; niacin, 24.5 mg; pyridoxine, 5 mg; biotin, 1 mg; folic acid, 1 mg; choline, 500 mg; Mn, 60 mg; I, 0.4 mg; Fe, 80 mg; Cu, 8 mg; Se, 0.3 mg; Zn, 60 mg.

^2^Crude protein, crude fiber, crude ash, and ether extract were measured values; others were based on calculated values.

The housing arrangement consisted of four hens per stainless steel cage, with cage dimensions measuring 40 cm (width) × 45 cm (length) × 45 cm (height). Each replicate comprised four of these cages, providing a stocking density of 450 cm^2^ per hen. The hens had *ad libitum* access to both feed and water. Lighting conditions adhered to the recommended cycle of 16 h of light followed by 8 h of darkness throughout the experimental period, supplemented by artificial lighting (incandescent lamps with a light intensity of 30 Lx). Room temperature was maintained within the range of 15–22°C, and relative humidity was kept between 70 and 80%. An initial week was designated as an adaptation period, followed by 7 weeks allocated to the formal experiment.

### 2.4 Sample collection

At the conclusion of the formal experiment, 12 hens of moderate weight from each group were randomly selected (2 hens per replicate) and subjected to a 12-h fasting period. Subsequently, the selected 48 hens underwent blood sample collection. Blood samples (5 mL) were drawn from the brachial vein into a 10 mL anticoagulant-free Vacutainer tube (Greiner BioOne GmbH, Kremsmünster, Austria). After standing for 2 h at 37°C, the collected blood samples were centrifuged at 3,000 rpm for 15 min at 4°C to extract the serum, which was then stored at −80°C. Following blood collection, the hens were euthanized via cervical dislocation. The feathers were removed, and the hens were weighed to determine slaughter weight. The liver and spleen were subsequently isolated, weighed, and immediately stored at −80°C. The relative weights of the liver and spleen were calculated as a percentage of the slaughter weight. Within 20 min post-slaughter, approximately 4 g of cecal content samples were swiftly collected, frozen, and preserved in liquid nitrogen at −80°C for subsequent 16S rRNA sequencing and untargeted metabolomic analysis.

### 2.5 Egg quality

A total of 36 eggs were randomly collected from each group (6 eggs per replicate) at the end of the trial period for further egg quality determination. Eggshell strength, haugh unit, and albumen height were measured utilizing a digital egg tester (DET-6000, Nabel Co., Ltd., Kyoto, Japan). In addition, physical measurements were conducted by determining the short axis and long axis of the eggs using a digital caliper with a sensitivity of 0.01 mm. The egg shape index was then calculated as the ratio of the long axis to the short axis.

### 2.6 Antioxidant capacity

Each liver sample was accurately weighed to 0.2 g, followed by the addition of a dilution solution with a volume nine times that of the sample weight. The samples were homogenized in an ice bath using a fast homogenizer for approximately 1 min, until the solution appeared free of visible particles. The homogenized mixture was then centrifuged at 3,000 rpm for 10 min at 4°C to remove any debris. A portion of the resulting supernatant was reserved for analysis, while the remaining solution was promptly stored at −80°C for subsequent examination.

The activities of total antioxidant capacity (T-AOC), total superoxide dismutase (T-SOD), and glutathione peroxidase (GSH-Px), along with the content of malondialdehyde (MDA) in both serum and liver samples, were measured using commercial kits obtained from Nanjing Jiancheng Institute of Bioengineering (Nanjing, China).

### 2.7 Amino acid profile in egg yolk

For amino acid analysis, 30 mg of each sample was homogenized and transferred to a sealed tube containing 10 mL of 6 mol/L HCl. The samples were then rapidly frozen in liquid nitrogen for 5 min before undergoing vacuum drying. Subsequently, the samples were subjected to post-column derivatization with ninhydrin and hydrolyzed in a thermostated container at 110°C for 22 h using a 0.1% phenol solution. Once the samples cooled to room temperature, the resulting solution was filtered into a 50 mL volumetric flask, and the final volume was adjusted with distilled water. Next, 1 mL of the filtrate was placed into a separate tube and dried under reduced pressure at 50°C. The resulting residue was re-dissolved in 2 mL of water and dried under reduced pressure once more. Finally, the samples were dissolved in 2 mL of sodium citrate solution, adjusted to a pH of 2.2, and analyzed for amino acid content using a Hitachi 835-50 amino acid automatic analyzer.

### 2.8 16s rRNA sequencing

The cecum content samples were carefully transferred into Eppendorf tubes and promptly preserved at −80°C using liquid nitrogen until further analysis. Subsequently, 16S rRNA sequencing was conducted following the methods outlined in our previously published research (Liu et al., 2023).

DNA extraction and PCR amplification were performed following the manufacturer’s guidelines. The Omega Bio-tek DNA Kit (Norcross, GA, United States) was used to extract microbial DNA from cecal content samples. The quality and concentration of the extracted DNA were assessed via 1.0% agarose gel electrophoresis and quantified with a ND-2000 spectrophotometer (Thermo Scientific Inc., United States). The V3-V4 hypervariable region of the bacterial 16S rRNA gene was amplified using the primer pairs 341F (5’-CCTAYGGGRBGCASCAG-3’) and 806R (5’-GGACTACHVGGGTWTCTAAT-3’) on an ABI PCR thermocycler (CA). The amplified products were analyzed on a 2% agarose gel, purified with the DNA Gel Extraction Kit (Axygen Biosciences, Union City, CA, United States), and quantified using a fluorometer (Promega). Sequencing was conducted on an Illumina MiSeq platform (Illumina, San Diego, CA) at Majorbio Bio-Pharm Technology Co., Ltd., Shanghai, China.

For sequence processing and analysis, demultiplexing was initially performed, followed by quality filtering using fastp (version 0.19.6) and merging with FLASH (version 1.2.11). High-quality sequences were denoised through the DADA2 plugin within the Qiime2 pipeline (version 2020.2) using recommended settings. This methodology enabled single-nucleotide resolution based on error profiles, resulting in the generation of amplicon sequence variants (ASVs) through the DADA2 process. Taxonomic classification of the ASVs was carried out using the Naive Bayes consensus taxonomy classifier in Qiime2, referencing the SILVA 16S rRNA database (v138).

### 2.9 Untargeted metabolomic analyses

The metabolites were extracted from 50 mg of cecal content samples by accurately weighing them and then using a 400 μL methanol: water (4:1) solution with 0.02 mg mL^–1^ L-2-chlorophenylalanine as an internal standard. The mixture was allowed to settle at −10°C and then treated using a high throughput tissue crusher Wonbio-96c (Shanghai Wanbo Biotechnology Co., Ltd.) at 50 Hz for 6 min, followed by ultrasound at 40 kHz for 30 min at 5°C. Subsequently, the samples were placed at −20°C for 30 min to precipitate proteins. After centrifugation at 13,000 g at 4°C for 15 min, the supernatant was carefully transferred to sample vials for LC-MS/MS analysis.

The raw LC/MS data was preprocessed using Progenesis QI software (Waters Corporation, Milford, USA), which exported a three-dimensional data matrix in CSV format. Each ion was identified based on a combination of retention time and m/z data. Online databases such as KEGG and HMDB were utilized to annotate the metabolites by matching the exact molecular mass data (m/z) from the samples with entries in the databases. Metabolic features detected in at least 80% of any set of samples were retained. Following filtering, minimum metabolite values were imputed for specific samples where metabolite levels fell below the lower limit of quantitation, and each metabolic feature was normalized by sum. Variance analysis was then performed on the matrix file after data preprocessing. The R package ropls (Version 1.6.2) conducted principal component analysis (PCA) and orthogonal partial least squares discriminant analysis (OPLS-DA), with 7-cycle interactive validation used to assess model stability. The student’s *t*-test and fold difference analysis were employed. Significantly different metabolites were selected based on the Variable Importance in Projection (VIP) obtained from the OPLS-DA model and the *p*-value from student’s *t*-test. Metabolites with VIP > 1 and *P* < 0.05 were considered significantly different.

### 2.10 Statistical analysis

The experimental data was subjected to analysis of variance (ANOVA) followed by Tukey’s *post hoc* test for multiple comparisons using SPSS 20.0 software (IBM-SPSS Inc., United States). The results were presented as means with the Standard Error of the Mean (SEM), and statistical significance was accepted at a value of *P* < 0.05. GraphPad Prism 8.0.2 software (Monrovia, CA, United States) was utilized for data visualization and further statistical analysis. Given the non-linear distribution of microbial relative abundance, Spearman correlations were employed to examine the associations between microbiota and metabolites, as well as egg quality, amino acids in yolk and antioxidant indicators. The Pearson correlation analysis was conducted to assess the correlations between metabolites and nutrient, as well as antioxidant indicators data.

## 3 Results

### 3.1 Carcass characteristics and egg quality

The carcass characteristics and egg quality of hens fed various diets are presented in Supplementary Figure S1. Our results indicate that, compared to the CON group, the laying hens in the T150 group achieved the highest slaughter weight throughout the study. In contrast, increasing multi-enzyme supplementation had no effect on the percentages of liver or spleen.

Some data of egg quality were from our previous study (Huang et al., 2024). Compared to the CON group, the egg shape index in the T150 group was significantly increased (*P* < 0.05). The albumen height and Haugh units in T600 group were significantly higher than those in the CON group (*P* < 0.05). Our results also indicated an upward trend in the egg shape index, albumen height, and haugh units with the inclusion of multi-enzyme supplementation in the diet. However, we found that the novel multi-enzyme supplementation had no significant effects on eggshell strength and average egg weight. Other production performance had been also assessed and published in our previous research (Huang et al., 2024).

### 3.2 Antioxidant parameters

The effects of different levels of novel multi-enzyme on antioxidant parameters of laying hens are depicted in Table 2. With increasing levels of multi-enzyme supplementation in the diet, total antioxidant capacity (T-AOC) activity in the serum was significantly increased (*P* = 0.005). Similarly, the activity of glutathione peroxidase (GSH-Px) in the liver was significantly enhanced (*P* = 0.010) with higher multi-enzyme supplementation. Compared to the CON group, the T600 group showed the highest improvement in the T-AOC activity in serum and GSH-Px activity in liver. However, no significant differences were observed in malondialdehyde (MDA), superoxide dismutase (SOD), and GSH-Px levels in the serum, or in MDA, T-AOC, and SOD levels in the liver across the various multi-enzyme supplementation levels.

**TABLE 2:** Effects of multi-enzyme on antioxidant capacity of laying hens.

Items	CON	T150	T300	T600	SEM	*P*-value
**Serum**						
MDA, nmol mL^–1^	7.00	2.41	3.51	5.04	0.77	0.172
T-AOC, U mL^–1^	0.58^b^	0.63^b^	0.70^ab^	0.83^a^	0.03	0.005
SOD, U mL^–1^	141.59	144.88	147.6	147.98	1.145	0.170
GSH-Px, U mL^–1^	2748	2928	3216	3384	136.166	0.379
**Liver**						
MDA, nmol mg prot^–1^	0.35	0.33	0.28	0.31	0.019	0.627
T-AOC, U mg prot^–1^	0.91	0.96	1.09	0.94	0.030	0.131
SOD, U mg prot^–1^	54.35	58.50	64.69	75.44	4.865	0.471
GSH-Px, U mg prot^–1^	128.23^c^	136.69^c^	153.66^b^	159.33^a^	2.090	0.010

Data with different superscripts in the same row are different significantly (*P* < 0.05). MDA, malondialdehyde; T-AOC, total antioxidant capacity; SOD, superoxide dismutase; GSH-Px, glutathione peroxide.

### 3.3 Amino acid profiles in egg yolks

Table 3 detailed the influence of multi-enzyme supplementation on amino acid profiles within the egg yolk. There were significant effects of multi-enzyme supplementation on serine, phenylalanine, tyrosine, glutamate, proline and isoleucine content in egg yolk. Specifically, the content of serine gradually increased in T150 and T300 groups, but was significantly lower in T600 group than in T150 and T300 groups. Compared to the CON group, the content of phenylalanine, tyrosine and isoleucine in T150 group were significantly increased, which increased by 16.44, 11.76, and 9.10%, respectively. Additionally, the content of glutamate and proline in T2 group were also significantly enhanced, which enhanced by 14.01 and 12.9% respectively, compared with the CON group. Conversely, no significant differences were observed in other amino acids among treatment groups and the CON group.

**TABLE 3:** Effects of multi-enzyme on amino acid profiles in yolk of laying hens.

Items	CON	T150	T300	T600	SEM	*P*-value
Aspartate, g 100g^–1^	1.45	1.43	1.49	1.36	0.031	0.560
Threonine, g 100g^–1^	0.79	0.84	0.82	0.79	0.011	0.248
Serine, g 100g^–1^	1.19^ab^	1.29^a^	1.31^a^	1.11^b^	0.029	0.024
Arginine, g 100g^–1^	1.18	1.02	1.19	1.11	0.064	0.809
Histidine, g 100g^–1^	0.42	0.45	0.42	0.40	0.008	0.109
Phenylalanine, g 100g^–1^	0.73^b^	0.85^a^	0.73^b^	0.72^b^	0.016	<0.001
Lysine, g 100g^–1^	1.25	1.34	1.30	1.25	0.018	0.208
Tyrosine, g 100g^–1^	0.68^b^	0.76^a^	0.71^ab^	0.69^b^	0.011	0.021
Leucine, g 100g^–1^	1.43	1.53	1.44	1.38	0.023	0.116
Glutamate, g 100g^–1^	1.57^ab^	1.60^ab^	1.79^a^	1.36^b^	0.058	0.037
Proline, g 100g^–1^	0.62^b^	0.67^ab^	0.70^a^	0.66^ab^	0.010	0.033
Isoleucine, g 100g^–1^	0.88^b^	0.96^a^	0.89^ab^	0.88^b^	0.012	0.027
Methionine, g 100g^–1^	0.43	0.45	0.41	0.39	0.008	0.087
Valine, g 100g^–1^	0.96	1.04	1.01	0.99	0.012	0.115
Alanine, g 100g^–1^	0.78	0.79	0.83	0.77	0.016	0.627
Glycine, g 100g^–1^	0.47	0.48	0.49	0.45	0.009	0.537

Data with different superscripts in the same row are different significantly (*P* < 0.05).

### 3.4 The microbial profiles of the cecum

**FIGURE 1:**
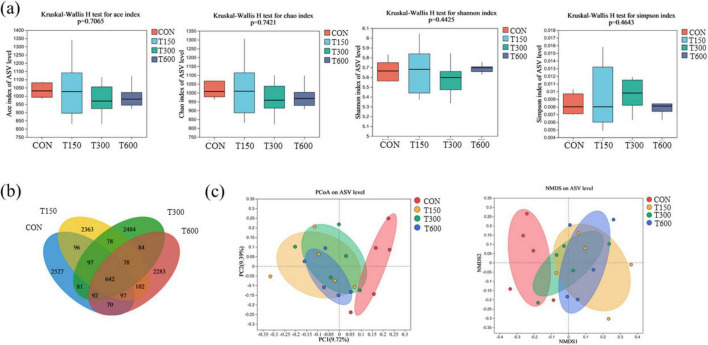
Effects of multi-enzyme in feed on diversity analyses of microbial communities among groups. **(A)** Alpha diversity (Ace, Shannon, Simpson, and Chao); **(B)** Venn diagram presenting the amplicon sequence variants (ASVs) from each group; **(C)** principal coordinates analysis (PCoA) and non-metric multidimensional scaling analysis (NMDS) of microbial communities among groups based on Bray–Curtis distance. CON, 0 mg kg^–1^ multi-enzyme; T150, 150 mg kg^–1^ multi-enzyme; T300, 300 mg kg^–1^ multi-enzyme; T600, 600 mg kg^–1^ multi-enzyme.

**FIGURE 2:**
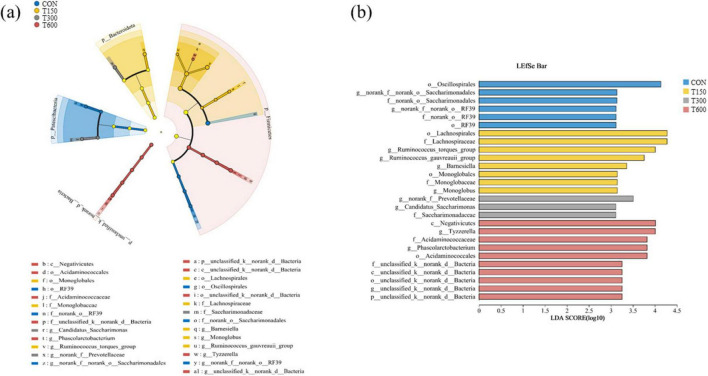
Linear discriminant analysis (LDA) effect size (LEfSe) analysis of the cecum microbial community in the four groups. **(A)** The cladogram of LEfSe analysis; **(B)** the histogram of LEfSe analysis. p_, phylum level; c_, class level; o_, order level; f_, family level; g_, genus level; CON, 0 mg kg^–1^ multi-enzyme; T150, 150 mg kg^–1^ multi-enzyme; T300, 300 mg kg^–1^ multi-enzyme; T600, 600 mg kg^–1^ multi-enzyme.

In this trial, all treatment groups had no significant effect (*P* > 0.05) on the alpha diversities (ACE, Simpson, Shannon and Chao) of the microbiota in cecum of laying hens compared to the CON group (Figure 1A). A total of 11,104 amplicon sequence variants (ASVs) were identified across the four groups, with the numbers of unique ASVs being 2,527, 2,363, 2,484, and 2,283, respectively. The common ASVs among the four groups totaled 642 (Figure 1B). Beta diversity, as illustrated in the PCoA and NMDS scatterplot, indicated a significant shift in the treatment groups (T150, T300, and T600) compared to the control group, with notable overlap among the three treatment groups (Figure 1C). The LEfSe analysis further highlighted significant differences in the relative abundance of bacteria in the cecal microbiota among the four groups. Bacterial taxa with LDA score larger than 3 were selected as biomarker taxa. The analysis (LDA score > 3) revealed 27 taxa biomarkers across the four groups, which mainly belong to the phylum of *Bacteroidota*, *Patescibacteria* and *Firmicutes*, the class of *Negativicutes*, the order of *Oscillospirales*, *Saccharimonadales*, *RF39*, *Lachnospirales*, *Monoglobales*, and *Acidaminococcales*, and the family of *Lachnospiraceae* and *Acidaminococcaceae* (Figures 2A, B).

**FIGURE 3:**
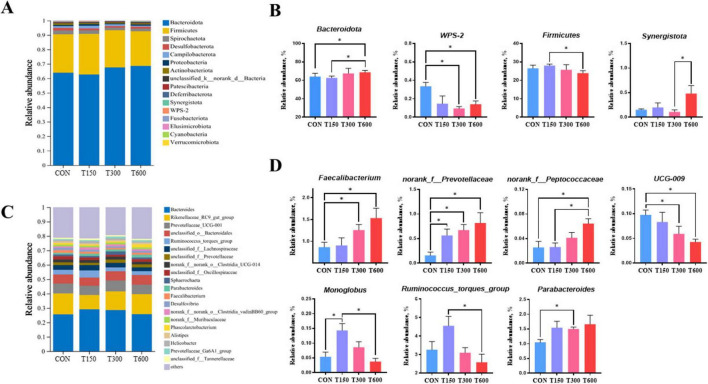
Effects of multi-enzyme in feed on cecal microbial communities. **(A)** Average relative abundance of microbial species in the cecum at the phylum level; **(B)** relative abundance of microbial communities in the cecum contents at the phylum level; **(C)** average relative abundance of microbial species in the cecum at the genus level; **(D)** relative abundance of microbial communities in the cecum contents at the genus level; Data are expressed as mean ± standard error of mean (SEM) (*n* = 5); Significant differences are indicated by “*” (*P < 0.05). CON, 0 mg kg^–1^ multi-enzyme; T150, 150 mg kg^–1^ multi-enzyme; T300, 300 mg kg^–1^ multi-enzyme; T600, 600 mg kg^–1^ multi-enzyme.

Moreover, the relative abundances of microbiota at the phylum and genus levels were also performed and displayed in Figure 3. The relative abundances of bacteria in cecum at the phylum level was showed in Figure 3A, with *Firmicutes* and *Bacteroidota* were main phyla. We found that there was four notably different microbiota at the phylum level among the four groups (Figure 3B). The relative abundance of the *Bacteroidota* phyla in T600 group was significantly increased (*P* < 0.05) compared to CON and T150 groups. The relative abundance of the *Firmicutes* phylum in T600 group were significantly lower than T150 group (*P* < 0.05). At the genus level, we analyzed the microbiota composition of the top 20 genera (Figure 3C) and identified seven genera with significantly different abundances among the four groups (Figure 3D). The relative abundance of the *Parabacteroides* genera in T300 group, the *Faecalibacterium* genera in T300 and T600 groups, the *norank_f_Prevotellaceae* genera in treatment groups (T150, T300 and T600), the *norank_f_Peptococcaceae* genera in T600 group, and the *Monoglobus* genera in T150 group were significantly increased. Conversely, the relative abundance of the *UCG-009* genera in T300 and T600 groups were significantly decreased compared to the CON group. In addition, the relative abundance of the *Monoglobus* and *Ruminococcus_torques_group* genera in T600 group were significantly reduced, compared with the T150 group. As for the relative abundance of *Faecalibacterium* genera, T300 group was significantly lower than in the T150 group.

### 3.5 The metabolic profiles of the cecum

**FIGURE 4:**
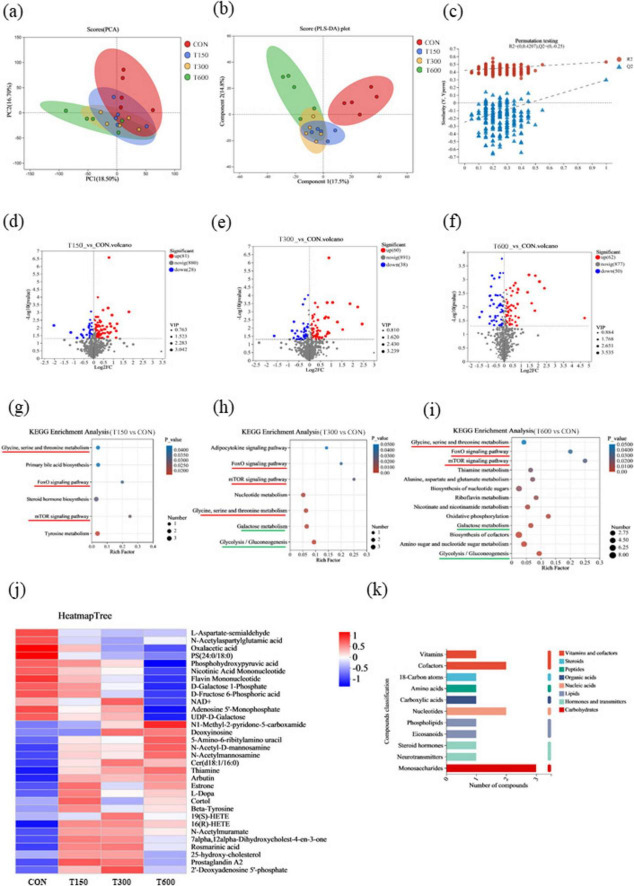
Effects of multi-enzyme in feed on the metabolite profiles in the cecum. **(A)** Principal component analysis (PCA) score plot of non-targeted metabolite profiling of the cecal samples among groups; **(B)** score plots from the partial least-squares discriminant analysis (PLS-DA) model among groups; **(C)** PLS-DA substitution test; **(D–F)** Differential metabolite volcano plots; **(G)** KEGG enrichment analysis of differential metabolites identified in T1 and CON groups; **(H)** KEGG enrichment analysis of differential metabolites identified in T2 and CON groups; **(I)** KEGG enrichment analysis of differential metabolites identified in T3 and CON groups; **(J)** the union of significantly different metabolites from each treatment group vs CON group (32 metabolites) were selected according to *P*-values < 0.05, and the differences were shown in the heatmap; **(K)** classification of KEGG compounds of 32 metabolites. Shade of color represents the differences, while bubble size represents the number of enrichments. CON, 0 mg kg^–1^ multi-enzyme; T150, 150 mg kg^–1^ multi-enzyme; T300, 300 mg kg^–1^ multi-enzyme; T600, 600 mg kg^–1^ multi-enzyme.

To explore the metabolic profiles and metabolic pathways induced by novel multi-enzyme supplementation, an LC-MS/MS-based untargeted metabolomic analysis was used to determine cecum metabolite profiles. The PCA showed 18.5 and 16.7% variances by PC1 and PC2, respectively, with partial overlap between the CON and three treatment groups in score plots (Figure 4A). But the PLS-DA plot displayed class-discriminating variations, which showed that a clear separation between the CON and treatment groups, with overlap among three treatment groups (Figure 4B). The results showed that the intercept between the regression line of Q2 and vertical axis is <0.05, suggesting successful model replacement and the model conformed to the real situation of sample data (Figure 4C). In this study, a total of 109 metabolites in the cecal were identified and considered as differential biomarkers between T150 and CON group, of which 81 up-regulated and 28 down-regulated metabolites (Figure 4D). Similarly, a total of 98 differential expression metabolites in the cecal were identified between T300 and CON group, of which 60 up-regulated and 38 down-regulated metabolites (Figure 4E). Between T600 and CON group, there were 112 differential expression metabolites in the cecal were identified, among which 62 up-regulated and 50 down-regulated (Figure 4F).

In order to further explore the specific effects of different levels of novel multi-enzyme supplementation on metabolic pathways, we performed KEGG pathway analysis to annotate the metabolic pathways involved. The enrichment pathways (*P* < 0.05) between each treatment group and CON group were shown in Figures 4G–I. The common enrichment metabolic pathways of each treatment group compared to the CON group were glycine, serine and threonine metabolism, foxo signaling pathway and mTOR signaling pathway (Figures 4G–I). And the enrichment metabolic pathways shared by T300 vs CON and T600 vs CON was galactose metabolism and glycolysis/gluconeogenesis pathways (Figures 4H, I). The union of significantly different metabolites from each treatment group vs CON group were selected according to *P*-values < 0.05, and the differences were shown by heatmap (Figure 4J). Compared with the CON group, of the 12 significantly different down-regulated molecules, most showed lowest abundances in the T600 group. Among the rest of 20 significantly different up-regulated molecules compared with the CON group, most of them show no notably difference among three treatment groups (Figure 4J). KEGG compound classification was performed on 32 metabolites, and the results are shown in Figure 4K. Three monosaccharides were identified, including n-acetyl-D-mannosamine, n-acetylmuramate and n-acetylmannosamine.

### 3.6 Correlation analysis

**FIGURE 5:**
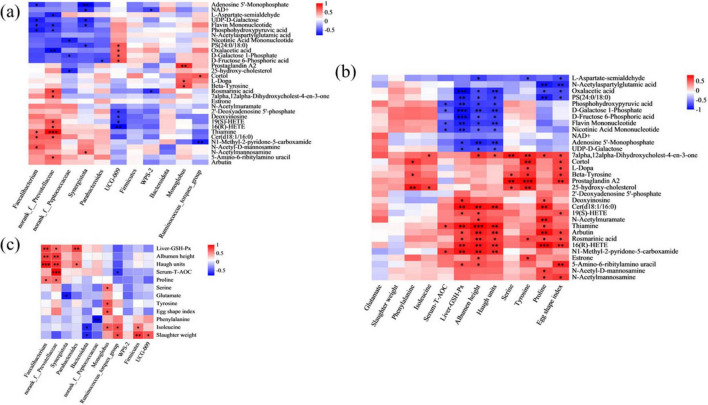
Heatmaps of correlation analysis. **(A)** Correlation between differential microbiota and differential metabolites; **(B)** correlation among egg quality, amino acids in yolk and antioxidant capacity indicators and differential metabolites; **(C)** correlation among egg quality, amino acids in yolk and antioxidant capacity indicators and differential microbiota. Shade of color represents the differences. Significant correlations are indicated by “*”, “**” and “***” (*P < 0.05, **P < 0.01, and ***P < 0.001).

The correlations among metabolites in cecum, cecal microbes and indicators (egg quality, nutrients in yolk and antioxidant capacity) were examined to further confirm the underlying mechanisms. The spearman correlation depicted in Figure 5A revealed that the abundance of *norank_f_Prevotellaceae* genera was positively correlated with thiamine, cer(d18:1/16:0), 19(S)-HETE, rosmarinic acid, 16(R)-HETE, 7alpha,12alpha-dihydroxycholest-4-en-3-one and 5-amino-6-ribitylamino uracil, and negatively correlated with flavin mononucleotide, oxalacetic acid, phosphohydroxypyruvic acid and L-aspartate-semialdehyde. Moreover, the abundance of *Parabacteroides* genera was negatively correlated with D-fructose 6-phosphoric acid, and the abundance of *Monoglobus* genera was positively correlated with L-dopa, beta-tyrosine and prostaglandin A2. The abundance of *Ruminococcus_torques_group* genera was positively correlated with cortol and negatively correlated with N1-methy1-2-pyridone-5-carboxamide. The Figure 5B showed that T-AOC in serum, GSH-Px in liver, albumen height and haugh units were positively correlated with N1-methy1-2-pyridone-5-carboxamide and thiamine, and negatively correlated with phosphohydroxypyruvic acid, D-galactose 1- phosphate, flavin mononucleotide and nicotinic acid mononucleotide. Additionally, the content of serine, tyrosine and proline in egg yolk were positively associated with 7alpha,12alpha-dihydroxycholest-4-en-3-one, and the content of phenylalanine and isoleucine in egg yolk were positively associated with 25-hydroxy-cholesterol. As shown in the Figure 5C, the abundance of *norank_f_Prevotellaceae* genera was positively associated with the activities of T-AOC in the serum, GSH-Px in the liver, the content of proline in yolk and albumen height and haugh units. In addition, the abundance of *Parabacteroides* genera was positively associated with the activity of GSH-Px in the liver and haugh units, the content of serine, tyrosine, and isoleucine in yolk was positively associated with *Monoglobus* genera, and the content of phenylalanine and glutamate in yolk were negatively associated with *norank_f_Peptococcaceae* genera and *Synergistota* phylum respectively.

## 4 Discussion

Enzymes have been widely utilized as feed supplements in laying hens, with previous studies primarily focusing on their impacts on performance and egg quality (Olgun et al., 2018; El-Hack et al., 2019). However, there is a dearth of research exploring the effects of enzyme application on amino acid profiles in yolk, cecal microbiota structure, and metabolites of laying hens. The objective of this study was to determine the effects of the multi-enzyme (alkaline protease, xylanase, glucanase, β-mannanase, cellulase, acid protease, glucamylase and α-galactosidase) on antioxidant capacity, egg quality, amino acid profiles in yolk, cecal microflora and metabolites.

The evaluation of egg quality is vital in poultry production, with key indicators including egg shape index, albumen height, haugh unit, and eggshell strength (Poudel et al., 2023). Consumer preferences often lean toward oval-shaped eggs, as indicated by pre-market surveys (Mitrovic et al., 2022). Research consistently demonstrates that higher albumen height and haugh unit are associated with improved albumen viscosity, enhanced egg quality, and a greater concentration of bioactive substances in the albumen (Geng et al., 2023; Zhang et al., 2023). In our study, we observed that the egg shape index, albumen height, and haugh unit were higher in all treatment groups (T150, T300, and T600) compared to the CON group. These results suggested that the addition of multi-enzyme to feed help to improve egg quality, which makes it easier to attract customers and enhances profitability. Our results align with previous research conducted by Van Hoeck et al. (2021), who reported a significant increase in albumen height and haugh unit in laying hens fed diets containing xylanase (at doses of 45,000 and 90,000 U kg^–1^) compared to the control group at week 12.

Amino acid profiles are widely recognized for their significant impact on food flavor, with various amino acids contributing to different taste perceptions, including sweetness, sourness, saltiness, bitterness, and umami (Dajnowska et al., 2023). Studies have reported that glutamate, aspartate, alanine and tyrosine contributed umami taste, and methionine, alanine, glycine, proline, serine, valine and lysine contributed sweet taste (Iwaniak et al., 2016). Our results revealed that the content of glutamate, tyrosine (contributing to umami taste), proline, and serine (contributing to sweet taste) were higher in groups supplemented with 150 and 300 mg kg^–1^ of multi-enzyme compared to the CON group. These findings suggest that the addition of multi-enzyme can alter the amino acid composition in the yolk, potentially influencing the taste profile of the eggs.

Enzymatic antioxidants such as T-AOC, SOD, and GSH-Px, along with MDA, a byproduct of lipid peroxidation, are crucial indices for evaluating antioxidant capacity in animals (Wu et al., 2024b). GSH-Px is essential for scavenging free radicals and converting hydrogen peroxide into water (Zhou et al., 2021). In our experiment, we observed an increase in T-AOC activity in serum and GSH-Px activity in the liver across all three treatment groups compared to the CON group. These findings suggest that dietary supplementation with multi-enzyme could enhance the antioxidant capacity in laying hens. Our results were in line with those of Liu Y. et al. (2021), who demonstrated that administration of 20 mg kg^–1^ of multi-enzyme (containing 11,000 U g^–1^ proteases, 20 U g^–1^ pullulanase, 1,000 U g^–1^ α-amylase, and 1,000 U g^–1^ glucoamylases) markedly increased serum T-AOC levels. Moreover, a previous study showed that supplementation with a novel protease significantly increased T-SOD and catalase activities in the serum of broilers (Yi et al., 2024c).

The gut microbiota plays a crucial role in intestinal health, nutrient absorption, and immune system regulation in animals. In our study, we observed that the relative abundance of *Bacteroidetes* and *Firmicutes* phyla in the T300 and T600 groups were slightly higher and lower, respectively, compared to the CON group. These trends are consistent with recent research reporting a higher proportion of *Bacteroidetes* and a lower proportion of *Firmicutes* in enzyme supplement treatment groups (Yi et al., 2024b). The ratio of *Firmicutes* to *Bacteroidetes* is often considered a biomarker of obesity, with obese animals typically exhibiting a high ratio (Grigor’eva, 2020; Magne et al., 2020). Recent studies have highlighted the beneficial roles of *Bacteroidetes*, including their production of B vitamins and propionate to enhance intestinal health and integrity (Yoshii et al., 2019; Zhang et al., 2019). Studies have found that an increase in the relative abundance of *Bacteroidetes*, accompanied by increased microbial community diversity, inhibits pathogen colonization while improving nutrient availability, thereby supporting increased egg production during the laying period (Dai et al., 2022). At the genus level, we observed a significant increase in the relative abundance of *Faecalibacterium* and *norank_f_Prevotellaceae* genera in the T300 and T600 groups. Cai et al. (2024) also found that the proportion of *Faecalibacterium* genera in 100 mg kg^–1^ protease DE200 treatment group was higher than that in control treatment. *Faecalibacterium* abundance is often associated with intestinal health, and low levels are linked to inflammatory diseases (Ferreira-Halder et al., 2017; Martín et al., 2023). As a major producer of short-chain fatty acids, *Faecalibacterium* exerts anti-inflammatory effects by metabolizing dietary fiber to provide an energy source for intestinal epithelial cells (Wang et al., 2021). Wang et al. (2020) indicated that the increase in the relative abundance of *Faecalibacterium* may modulate the immunity of breeding geese, thereby improving egg production performance. *Prevotellaceae* genera produce short-chain fatty acids and butyrate, which play essential roles in intestinal barrier integrity and homeostasis (Chen et al., 2022).

Our findings revealed that dietary supplementation with multi-enzyme significantly altered the metabolic profiles in the cecum of laying hens. The analysis of cecal metabolites demonstrated that the levels of three monosaccharides (n-acetyl-D-mannosamine, n-acetylmuramate, and n-acetylmannosamine) and thiamine were elevated in treatment groups (T150, T300, and T600) compared to the CON group. Cecal microbiota play a crucial role in hydrolyzing dietary polysaccharides into monosaccharides, which subsequently undergo catabolic processes to produce short-chain fatty acids. Short-chain fatty acids, in turn, protect the gut by providing energy and creating a low pH environment that inhibits pathogen growth (Krautkramer et al., 2021; Wang et al., 2023). Liu Y. S. et al. (2021) indicated that beneficial bacteria can promote short chain fatty acid production by fermenting polysaccharides, thus further enhancing host immunity and metabolism. Short chain fatty acid can be absorbed by the intestinal epithelial cells of animals as energy for growth and production, which in the cecum can be used as an indicator of gut health (Hu et al., 2018). Thiamine, also known as vitamin B1, is an essential metabolite produced by the microbial flora of the cecum. Once produced, it is transported through the intestinal mucosal cells into the bloodstream, where it is then distributed to tissues and cells throughout the body to exert its effects (Paez-Hurtado et al., 2023). Furthermore, our results indicated significant changes in the levels of adenosine 5′-monophosphate, 2′-deoxyadenosine 5′-phosphate, NAD^+^, flavin mononucleotide, and oxalacetic acid in treatment groups (T150, T300, and T600) compared to the CON group. These metabolites are products or intermediates involved in galactose metabolism and glycolysis/gluconeogenesis metabolism. L-DOPA (3,4-dihydroxyphenylalanine), an intermediate of tyrosine metabolism (Giuri et al., 2021) was upregulated in the treatment groups compared to the CON group. These findings suggested that multi-enzyme supplementation enhances the production of beneficial metabolites by the microbial flora in the cecum, thereby improving the cecal metabolism of laying hens.

In this study, correlation analysis revealed positive associations between albumen height, haugh unit, and liver GSH-Px levels with the *Faecalibacterium* genera. Similarly, these parameters were positively associated with thiamine levels. In addition, liver GSH-Px and haugh unit were found to be positively correlated with the *Parabacteroides* genus. Previous research has highlighted the protective effects of *Faecalibacterium* genera on digestive health, immune function, and metabolism (Effendi et al., 2022). Wu et al. (2024a) indicated the content of TC and LDL-C of ovary were negatively correlated with and *Faecalibacterium*, while the content of TP of ovary and egg yolk were positively correlated with *Faecalibacterium*. *Ruminococcus_torques_group* is a member of family with the ability to produce short chain fatty acids. Some studies have found that low abundance of *Ruminococcaceae* was related to inflammatory bowel diseases (Liu et al., 2022). Bai et al. (2023) found that *Ruminococcus_torques_group* was significant positively related to average egg weight, serum HDL-C level, GSH-Px activity, and shell strength, while was negatively correlated with haugh unit. This result was similar to our findings, which suggested that *Ruminococcus_torques_group* was negatively correlated with haugh units. *Prevotella* is a producer of short fatty acids, which belongs to the *prevotellaceae* family. Studies have found that *Prevotella* has the effect of improving glucose metabolism, probably by promoting the increase of glycogen storage (Kovatcheva-Datchary et al., 2015). Liu et al. (2020) found that *Prevotella_2* was significant positively related to the T-AOC in serum. Our results showed *norank_f_prevotellaceae* is positively related to the thiamine in the cecum and serum T-AOC, suggesting that *norank_f_prevotellaceae* plays a similar role to *prevotella* in the cecum of laying hens. *Parabacteroides* has been shown to mitigate metabolic disorders by influencing the production of succinic acid and secondary bile acids (Wang et al., 2019). Our findings suggest that the gut microbiota may enhance egg quality and antioxidant capacity by modulating metabolism, thereby promoting the overall health status of laying hens.

## 5 Conclusion

This study suggested multi-enzyme supplementation improved egg quality and antioxidant capacity, as well as altered amino acid profiles in yolk of laying hens. Furthermore, multi-enzyme supplementation in diet altered cecal microbial community and metabolites, resulting in increased levels of vitamin B1 and monosaccharides. Correlation analysis indicated that the observed changes in egg quality, yolk amino acid profiles, and antioxidant capacity following multi-enzyme supplementation were closely linked to the production and functions of specific bacteria and metabolites in the cecum.

## Data Availability

The datasets presented in this study can be found in online repositories. The names of the repository/repositories and accession number(s) can be found in this article/Supplementary material.

## Footnotes

1
http://en.sunhy.cn
