# Supplementary material for: Effects of various supplemental levels of multi-enzyme complex on amino acid profiles in egg yolk, antioxidant capacity, cecal microbial community and metabolites of laying hens
Source: Front Microbiol. 2024 Nov 28;15:1466024. doi: 10.3389/fmicb.2024.1466024 (PMC11634838; doi:10.3389/fmicb.2024.1466024)
Supplement: Supplementary file 1 [file Supplementary_file_1.docx]

Supplementary Material

# Supplementary Figures and Tables

## Supplementary Figures

**Supplementary Figure S1.** Effects of multi-enzyme in feed on the carcass characteristics and egg quality. (a) Carcass characteristics of laying hens. (b) Egg quality of laying hens. Data are expressed as mean ± standard error of mean (SEM) (n=12 or 6). Different lowercase letters indicate a significant difference (*P* < 0.05).
